# Iron affects localization of Ght5 in fission yeast

**DOI:** 10.1093/femsle/fnad022

**Published:** 2023-03-29

**Authors:** Sevim Nur Akyüz, Umit Yasar Kina, Ahmed S I Aly, Bedia Palabiyik

**Affiliations:** Department of Molecular Biology and Genetics, Institute of Graduate Studies in Sciences, Istanbul University, 34134, Istanbul, Turkey; Beykoz Institute of Life Sciences and Biotechnology, Bezmialem Vakıf University, 34820, Istanbul, Turkey; Beykoz Institute of Life Sciences and Biotechnology, Bezmialem Vakıf University, 34820, Istanbul, Turkey; Department of Biotechnology, Institute of Health Sciences, Bezmialem Vakif University, 34093, Istanbul, Turkey; Beykoz Institute of Life Sciences and Biotechnology, Bezmialem Vakıf University, 34820, Istanbul, Turkey; School of Science and Engineering, Al Akhawayn University, 53000, Ifrane, Morocco; Faculty of Science, Department of Molecular Biology and Genetics, Istanbul University, 34134, Istanbul, Turkey

**Keywords:** hexose transporters, Ght5, iron stress, glucose, membrane localization, *Schizosaccharomyces pombe*

## Abstract

Iron is an essential cofactor for eukaryotic cells, as well as a toxic metal under certain conditions. On the other hand, glucose is the preferred energy and carbon source by most organisms and is an important signaling molecule in the regulation of biological processes. In *Schizosaccharomyces pombe*, the Ght5 hexose transporter, known as a high affinity glucose transporter, is required for cell proliferation in low glucose concentrations. Herein, we aimed to investigate the effects of iron stress on the Ght5 hexose transporter under glucose repression and derepression conditions. The effect of iron stress on the expression profile of the *ght5* gene was analyzed by RT-qPCR and western blot. The localization of the Ght5-mNeonGreen fusion protein examined with confocal microscopy. Our results revealed that iron stress had an inhibitory effect on *ght5* expression, and it altered Ght5 localization on the cell surface, causing it to accumulate in the cytoplasm.

## Teaser

Effects of iron stress on Ght5, hexose transporter of *Schizosaccaromyces pombe*, under glucose repression and de-repression conditions were investigated.

## Introduction

Ferrous (Fe^+2^) and Ferric (Fe^+3^) states of iron, with the ability to gain and give electrons, acts as an electron transfer agent for many vital enzymes involved in reactions such as oxygen transport, activity of the tricarboxylic acid cycle, respiration, and replication (Kaplan and Kaplan 2009, Hentze et al. 2010, Cyert and Philpott 2013). But excess amounts of ferrous state can react with hydrogen peroxide, and form hydroxyl radical which is highly reactive and damaging to all kinds of molecules in cells such as sugars, amino acids, phospholipids, DNA bases, and organic acids (Halliwell and Gutteridge 1992; Schrettl et al. 2004 ).

Controlling iron concentrations is essential and critical for cells to respond rapidly to changes in extracellular iron levels (Askwith and Kaplan 1997). There are two different pathways for high-affinity iron uptake in the fission yeast *Schizosaccharomyces pombe*. First pathway is a reductive iron uptake system which employs a membrane bound ferrireductase (Frp1) enzyme, a cell surface multicopper oxidase (Fio1), and an iron permease (Fib1) (Roman et al. 1993). The second pathway involves hydroxamate-type siderophore biosynthesis, excretion, and transport of siderophore-ferric iron complexes (Miethke et al. 2007).

When iron is low, cells inhibit the expression of genes related to iron utilization with specific regulatory systems. Regulation in *S. pombe* is mediated at the transcriptional level through a heteromeric DNA binding complex (Mercier et al. 2006). These responses to changes in iron concentrations are to regulate the balance between available iron for essential biochemical reactions, and the accumulation of excess iron at deleterious levels (Labbe et al. 2007).

Glucose is the main source of energy and carbon for *S. pombe* as in other eukaryotes. Different phospho-signaling cascades appear to be used for proliferation and/or size control of *S. pombe* cells under low glucose conditions. One cascade includes Sds23-mediated down-regulation of the 2A-type protein phosphatases (PP2As) Ppa1, Ppa2, and the PP2A-like PP6 phosphatase, Ppel. Others are AMP-activated protein kinase pathway [Ssp2/AMPK or Ssp1/Ca2+/calmodulin-dependent kinase (CaMKK)], and TORC2, an evolutionarily conserved phosphatidylinositol 3-kinase-like protein kinase complex containing the target of rapamycin (TOR) kinase (Hanyu et al. 2009, Ikai et al. 2011, Toyoda et al. 2021).

Glucose uptake is mediated through a series of hexose transporters (Boles and Hollenberg 1997, Ozcan and Johnston 1999, Uldry and Thorens 2004, Manolescu et al. 2007). Hexose transporters are a well-defined family of proteins involved in cellular sugar uptake for a variety of organisms, including bacteria, yeast, plants, and humans (Heiland et al. 2000). Eight different hexose transporters (*ght1, ght2, ght3, ght4, ght5, ght6, ght7*, and *ght8*) have been identified in the *S. pombe* genome (Heiland et al. 2000, Saitoh et al. 2015). Expressions of *ght5, ght6*, and *ght8* increase under high glucose conditions, whereas expression of *ght5* only increases under low glucose conditions. Therefore, it has been suggested that *ght5* is sufficient and essential for cell proliferation under low glucose conditions (Saitoh et al. 2015).

There are limited studies on the relationship between *S. pombe* hexose transporters and abiotic stresses, while studies in *S. cerevisiae* show that different abiotic stresses cause changes in the expression of some hexose transporters (Türkel 1999, Liu et al. 2004, Angın 2017). In our previous studies, Ght5 hexose transporter's responses to oxidative stress and iron stress have been reported (Kina and Palabiyik 2019, Ozkan et al. 2019). In this study, we aimed to elucidate the response of Ght5 hexose transporter to iron stress at transcription, and translation levels in *Schizosaccharomyces pombe*.

## Materials and methods

### Strains, media, and culture conditions

Wild type *S. pombe 972h^−^*, and auxotrophic ED666 (ade6-M210, ura4-D18, leu1-32) as parental strain were used in this study. Cells (1 × 10^6^ mL^−1^) were grown in liquid Edinburgh Minimal Medium (EMM2) at 30°C with orbital shaking at 180 rpm. Medium was prepared with 0.67% yeast nitrogen base w/o amino acids, 0.5% or 2% glucose, and supplemented with adenine, histidine, leucine, lysine, and uracil at 50 µg/mL concentration (Petersen and Russell 2016). After transformation of *S. pombe* ED666 with plasmid containing uracil (Ura4^+^) gene as selection marker, selection media contained 2% glucose, and supplemented with only 50 µg/mL adenine and leucine. Plasmids used in this study were constructed, selected, and amplified by using *Escherichia coli* XL10-Gold (Agilent) cells. *E. coli* cells were grown in LB medium supplemented with 100 μg/mL ampicillin. All solid media contained 2% agar.

### Construction of plasmids and strains

**Figure 1.**
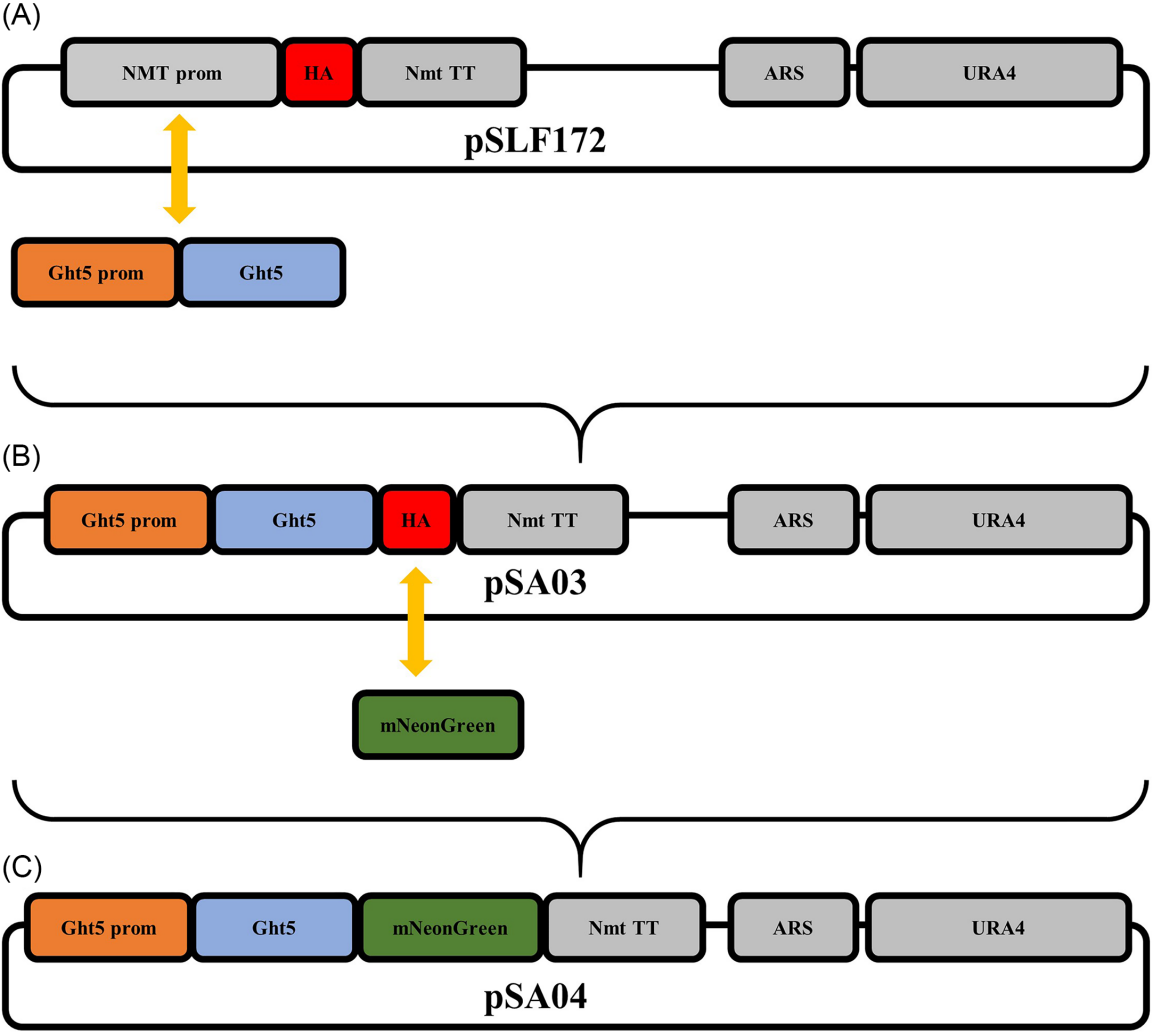
Schematic representation of the constructs. (**A**) pSLF172 is an episomal expression vector under the control of the nmt1 promoter, and has 3xHA epitope tag at carboxyl terminus. It also contains a yeast ARS and URA4 as selection marker (**B**) Ght5 was amplified with the primers designed to include its native promoter region. The 2.2 kb fragment was cloned into pSLF172 vector by replacing the nmt1 promoter, resulting plasmid (pSA03) was used for gene expression studies. (**C**) mNeonGreen fluorescent protein was amplified from commercially synthesized plasmid and tagged to the C terminus of *ght5* gene by replacing HA tag, resulting plasmid (pSA04) was used for localization studies. TT: transcription terminator, ARS:autonomously replicating sequence.

Genomic DNA was isolated from *S. pombe 972h^−^* as previously described (Looke et al. 2011), and used as a template to amplify a 2.2 kb fragment with Ght5_PstI_F (AACTGCAGCAGGTCTTGCGTCCAACTTT), and Ght5_NotI_R (ATAGTTTAGCGGCCGCGTGCGAATTGTTCTTCCTG) primers. The region targeted for amplification in PCR included the coding sequence of the *ght5* gene, and the region 600 bps upstream of the coding sequence that contains the gene's native promoter. Stop codon was not included. The fragment was digested with NotI and PstI restriction enzymes and inserted between the NotI and PstI enzyme sites of the *S. pombe* expression vector pSLF172 (Fig. 1A) (Forsburg and Sherman 1997), replacing the thiamine inducible nmt1 promoter. Resulting plasmid, pSA03, was verified by test digestion and sequencing. pSA03 (Fig. 1B), was transferred into *S. pombe* ED666 by electroporation method (Forsburg 2003). Briefly, yeast cells were grown untill OD_600_ reaches 0.5–1.5, and harvested at 4°C. Cells were washed with ice-cold 1 M sorbitol, then resuspended in 1 M sorbitol, and added into pre-chilled cuvettes. About 100 ng plasmid DNA was added to the cuvettes. Electroporation conditions were 1.5 kV, 200 Ω, 25 µF. Positive colonies were verified by colony PCR using primers Ght5_cds_F (TTTGGTGGTCTTTTCGTCCT), and NmtR (CGCACCCGTCTACGTTTCTA).

To generate ght5-mNeonGreen fusion gene, mNeonGreen sequence (Shaner 2013) was amplified from a commercially synthesized plasmid that contains mNeonGreen sequence by mNG_EagI_F (TGGGCGGCCGCATGGTAAGTAAGGGTGAAGAAGATAATATGGCT), and mNG_BamHI_R (GGCGGATCCTTTCTCGAATTGAGGATGACTCCAAGCACTACC) primers. The fragment was digested with EagI and BamHI restriction enzymes and inserted between the EagI and BamHI enzyme sites of pSA03 plasmid, replacing 3xHA epitope tag. Resulting plasmid, pSA04 (Fig. 1C), was verified by test digestion and sequencing. pSA04, was transferred into *S. pombe* ED666 as described above. The positive colonies were verified by colony PCR using primers Ght5_cds_F and NmtR.

### Determination of IC_50_ of iron

*S. pombe* parental (ED666), empty plasmid control (carrying pSLF172), and transformant SA03 (carrying pSA03 plasmid) strains have been used for the determination of 50% inhibitory concentration (IC_50_) of iron in liquid media. To perform the assay, the liquid cultures in EMM2 (2% glucose) were cultivated until the mid-logarithmic phase, later these cultures were diluted to 10^6^ cell/mL in both EMM2 with 0.5% glucose and EMM2 with 2% glucose media. Both cell cultures in 0.5% and 2% glucose were exposed to 0, 0.0625, 0.125, 0.25, 0.5, 1, 2, 4, 8 mM of FeCl_3 _× 6H_2_O (Merck). This experiment was performed in 96-wells microtiter plates, and the final volume was arranged to 200 μL per well. Cultures were grown at 30°C with orbital shaking at 180 rpm for 2 days. The optical densities of the cells were measured on a 96 wells plate reader at 600 nm (OD_600_) at 0th, 18th, 24th, 42nd, and 48th hours of growth. The IC_50_ value of iron for all cells was calculated according to the OD of the cultures at 24th hour. In statistical analysis, one-way analysis of variance (ANOVA) at GraphPad Prism 9.3.1 software was used. Differences in mean values were significant when *P* < 0.05.

### Protein extraction and western blot analysis

*S. pombe* whole cell protein extracts were prepared as previously described (Grallert and Hagan 2017). 30 mL OD_600 _= 0.2–0.3 cells were collected by centrifuge for each sample then resuspended in 250 µL RIPA Buffer (140 mM NaCl, 25 mM Tris-HCl,1 mM EDTA, 1% NP-40, 0.1% SDS) containing 1 mM Phenylmethylsulfonyl Fluoride (PMSF, Thermo Fisher Scientific). Glass beads were used in lysis of the cells. Each sample was vortexed for 1 minute, followed by incubation on ice for 1 minute. This cycle was repeated 4 times. Supernatants were separated by centrifugation at   21.000 x *g* at 4°C for 20 min. Protein concentrations were measured by Pierce BCA Protein Assay Kit (Thermo Fisher Scientific). 20 μg of total protein was used in western blot for each sample. First, proteins were separated by SDS-polyacrylamide gel electrophoresis then transferred to Polyvinylidene Fluoride (PVDF) membrane and later blotted to detect HA epitope-tagged proteins. Antibodies were diluted for blotting with α-Tubulin primary antibody (1:4.000; Novus Biologicals), α-Tubulin secondary antibody ( 1:10.000; Novus Biologicals), HA Tag primary antibody (1:3.000; Novus Biologicals), HA Tag secondary antibody (1:12 .500; Thermo Fisher Scientific). Bands were detected by using enhanced chemiluminescence (ECL) kit (Thermo Fisher Scientific).

### Total RNA isolation and RT-qPCR analysis

10 mL OD_600 _= 0.2 cells were harvested for total RNA isolation. Cells were resuspended in 500 µL RNAzol RT (Molecular Research Center) and glass beads were added. Then samples were vortexed for 1 minute, followed by incubation on ice for 1 minute. This cycle was repeated 4 times. 200 µL RNase-free water was added to supernatant and incubated for 10 min at room temperature then centrifuged at 12 .000 x *g* for 10 minutes. Later, 1:1 isopropanol was added to supernatant and incubated for 10 min then centrifuged at 12.000 x *g* for 10 minutes. Finally, pellet was washed twice with 75% ethanol and resuspended with RNase-free water. Concentration was measured by Nanodrop™ 8000 (Thermo Scientific, USA) spectrophotometer.

Isolated RNAs were treated with DNase enzyme at 37°C for 1 hour. Later samples were incubated at 75°C for 10 min to inactivate DNAse enzyme. cDNA was synthesized from 1 μg of total RNA, using High-Capacity cDNA Reverse Transcription Kits (Applied Biosystems) according to the manufacturer's instructions.

RT-qPCR was performed with the iTaq Universal SYBR Green Supermix (Biorad) following the manufacturer's instructions. Briefly, the reaction mixtures consisting of 10 μL SYBR Green Supermix, forward and reverse primers (0.2 µM of each) and 0.1 µg cDNA (the synthetic first-strand cDNAs) were up to 20 µL with ultra-pure nuclease-free water. Primer sequences were ght5_qF: TTTGGTGGTCTTTTCGTCCT and ght5_qR: CCAACAGCTGCGTAAATGAA for *ght5*, and gpd3_qF: GGTGACAACCACTCCTCCAT and gpd3_qR: TCAACAACACGGTGGGAGTA for the house-keeping gene. The PCR conditions were 95°C for 10 min (pre-incubation), followed by 40 cycles of 95°C for 10 sec, 53°C for 10 sec, and 72°C for 20 sec. The final step included gradual temperature increase from 55°C to 95°C at the rate of 1°C/10 sec to enable melting curve data collection. A non-template control was run and serial dilutions (1, 1:10 and 1:100) of the house-keeping (glyceraldehyde 3-phosphate dehydrogenase, *gpd3*), and the target genes were included in every assay. Amplification specificity of each reaction was verified by melting curve analysis. Expression levels were normalized against the reference gene, *gpd3*. Relative gene expression levels were determined according to the method previously described (Pfaffl 2001).

Statistical analyses were performed using Tukey's multiple comparisons test of two-way ANOVA in GraphPad Prism 9.3.1 software. Results were considered significant when the difference between the groups with the Tukey's multiple comparisons test was *P* < 0.05.

### Localization study and microscopy

Cells were grown until mid-logarithmic phase. Then cells were collected, and inoculated into fresh EMM2 mediums (0.5% glucose) containing 0.5, 1, and 2 mM iron. Cultures were incubated at 30°C at 180 rpm for 4 hours. 1 mL of cell culture was harvested, and washed 2 times with PBS. Pellet was diluted with 20–30 µL PBS. 2 µL of the sample was examined directly with confocal microscopy (Leica SP8).

## Results and Discussion

### Verification of experimental model

**Figure 2.**
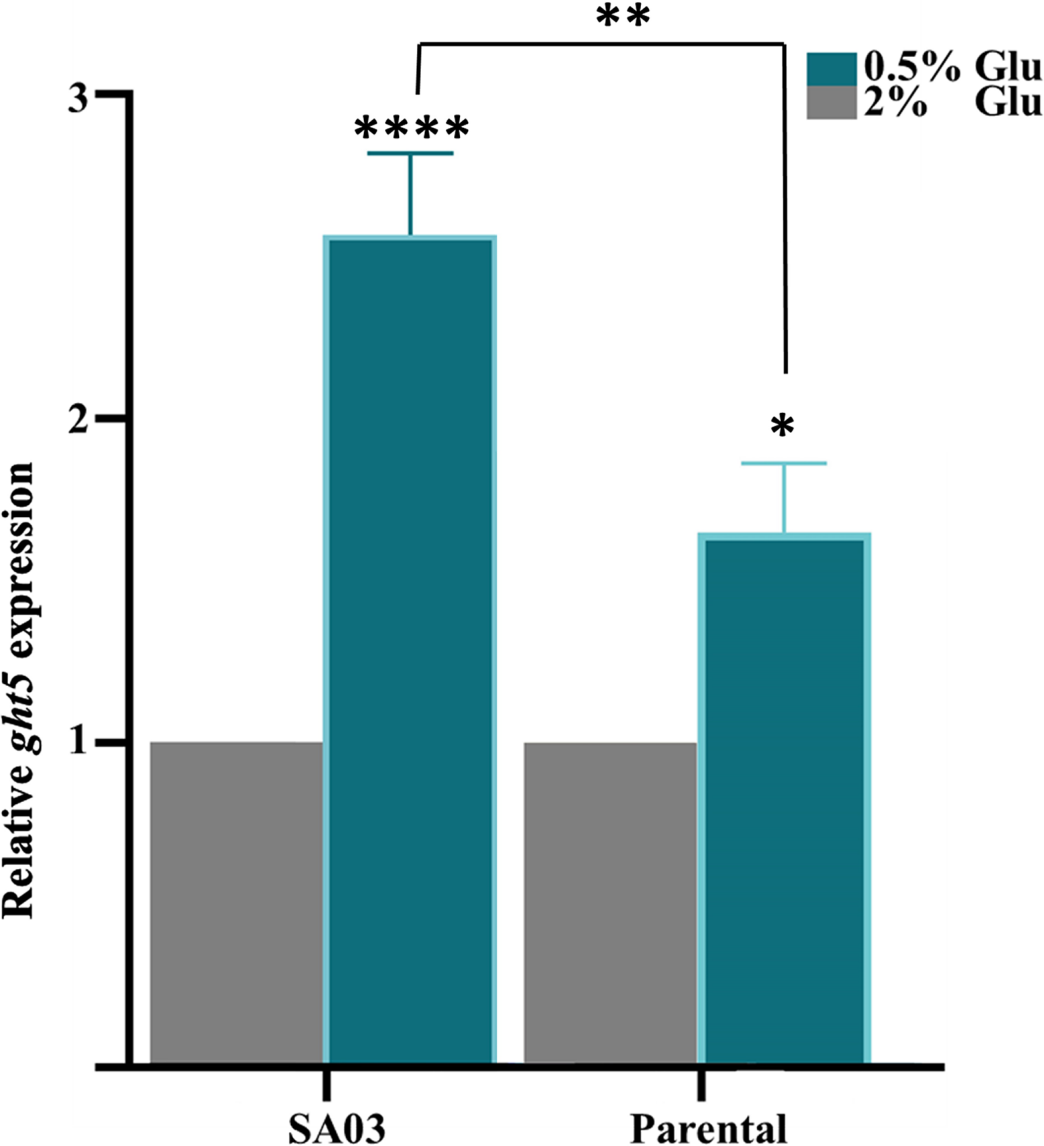
Relative expression of *ght5* in parental and SA03 cells under derepression condition. Transcript levels of hexose transporter *ght5* shows that repression of *ght5* is lifted under glucose starvation. Higher transcript levels in SA03 suggests that *ght5* promoter on episomal pSA03 plasmid is functional. *gpd3* gene was used as reference. Data represents mean value of three independent experiments. *P < 0.05, **P < 0.01, ^****^P < 0.0001

Ght5 expression is downregulated under glucose repression, but it is strongly expressed under 0.5% glucose (derepressed) condition (Heiland et al. 2000, Saitoh et al. 2015). Considering this, we tested the native *ght5* promoter of the pSA03 plasmid in SA03 cells by analyzing the relative expression of *ght5* under derepressed condition compared to repressed (2% glucose) condition. Also, *S. pombe* ED666 parental cells were tested at the same conditions. As expected, the expression level of *ght5* was increased in both cells under derepressed condition (Fig. 2). However, higher increase was observed in *ght5* expression in SA03 cells then parental cells due to multicopy expression of *ght5* gene from the episomal pSA03 plasmid alongside the native chromosomal locus. This result showed that the promoter in the pSA03 plasmid responds similarly to *ght5* regulation. Thus it is a convenient model system for studying effects of iron stress on *ght5* expression levels.

### Cytotoxic effect of iron on the growth of *S. Pombe* cells

Iron is an essential co-factor for eukaryotic cells, but it is also a toxic substance under specific conditions (Halliwell and Gutteridge 1992, Kaplan and Kaplan 2009, Hentze et al. 2010, Cyert and Philpott 2013; Markus 2004). To investigate the effects of iron stress on the hexose transporter, Ght5; we first determined the toxic iron concentrations for treatment on parental and SA03 cells.

**Figure 3.**
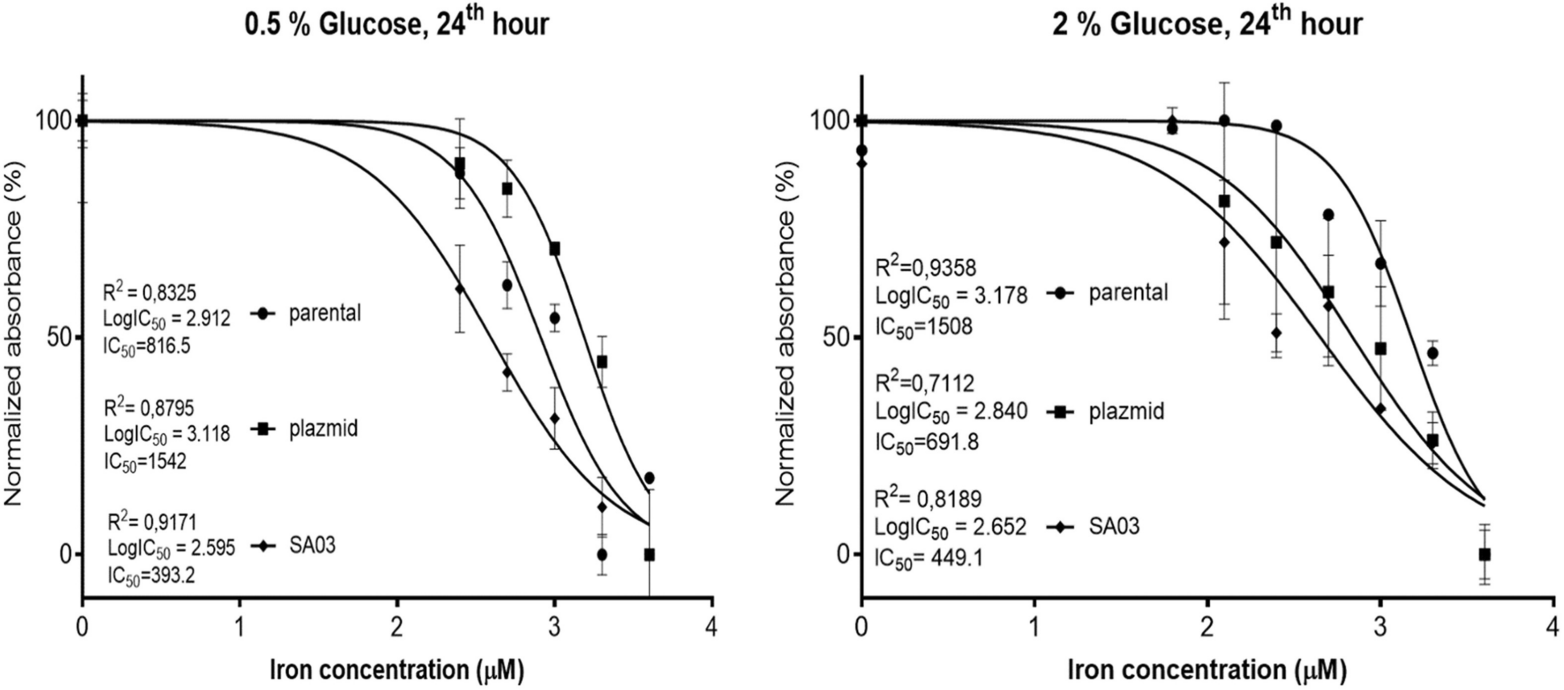
Analysis of iron toxicity. *S. pombe* ED666 parental cells, parental cells transformed with pSLF172 (empty plasmid), and SA03 were used. Cells were grown in 8 different concentrations of iron ranging from 62.5 µM to 8 mM. Cell growths were followed for 2 days by measuring OD_600_. IC_50_ of iron on cells was calculated by assessing their viability at 24th hour. Left: Cells were grown in EMM2 containing 0.5% glucose. Right: Cells were grown in EMM2 containing 2% glucose. Data represents mean value of three independent experiments.

The cytotoxic effect of iron on *S. pombe* ED666 parental, and SA03 cells were analyzed at different iron concentrations (62.5 µM to 8 mM) in minimal medium (EMM2) containing both 0.5% (repression) and 2% (derepression) glucose. We also included an empty plasmid control strain that carries pSLF172 plasmid to see the possible phenotypes due to the introduced plasmid itself. The toxic effect of iron was observed at ≥ 0.4 mM in both glucose conditions. Concentrations of iron higher than 2 mM was found to be lethal on all cells at both glucose conditions (Fig. 3). The inhibitory concentration of iron at which > 50% of the cells in liquid culture are inhibited (IC_50_) was calculated by assessing their viability at 24th hour. In all strains, the IC_50_ value was determined to be in the range between 0.4 mM-1.5 mM iron concentrations, in both glucose conditions. And 0.5, 1, and 2 mM concentrations were selected as iron stress conditions in the following experiments.

Results of empty plasmid and SA03 strains were inconsistent. We believe it is caused by instability of episomal plasmids in *S pombe*. Therefore we excluded transcription data of SA03 from the study. We had determined IC_50_ value of iron as 5 mM in our previous study (Ozkan et al. 2019). The reason for this difference might be due to the wild-type strain and the spot assay on YEA plates methods that were used in that study.

### Effects of iron stress on *ght5* transcription levels

Although there are many studies about the effects of abiotic stress conditions on *Saccharomyces cerevisiae* hexose transporters such as osmotic (Türkel 1999), iron (Wiśnicka et al. 1997), nickel and lead (Soares et al. 2003), arsenic (Liu et al. 2004), iron and cadmium (Angın 2017) these type of studies are limited in *S. pombe*. We previously showed relation between expression of hexose transporter Ght5 and oxidative stress (Kina and Palabiyik 2019), also iron stress (Ozkan et al. 2019) in *S. pombe*. In this study, we investigated the expression level of *ght5* gene at transcriptional and translational levels.

**Figure 4.**
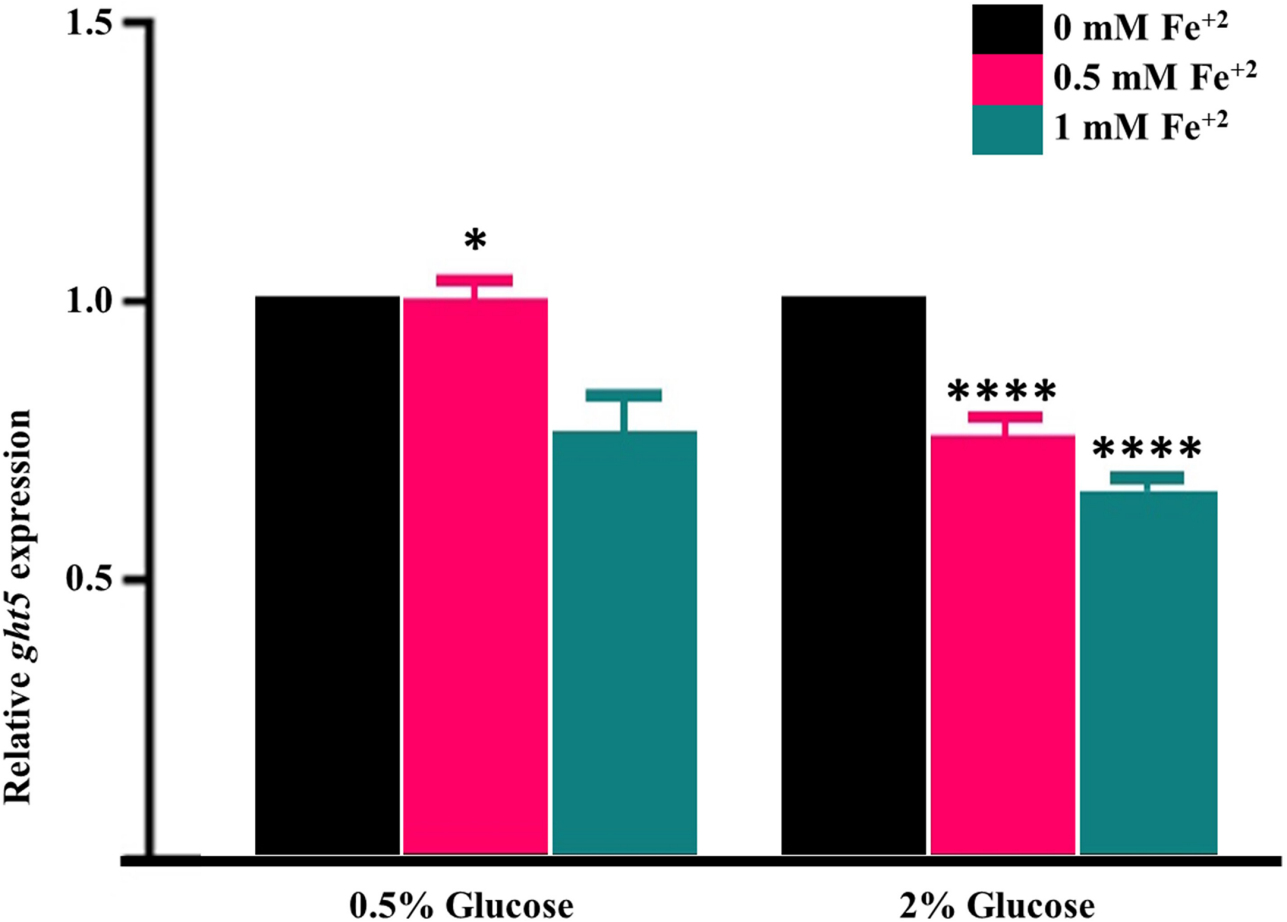
Relative expression of *ght5* under iron stress. RT-qPCR results of shows that iron stress inhibits *ght5* expression under both repressed and derepressed conditions. Iron-free (untreated) cultures were used as control. *gpd3* gene was used as reference. Data represents mean value of three independent experiments. *P < 0.05, ^****^P < 0.0001.

In *S. pombe* ED666 parental cells, RT-qPCR results show no significant change in the expression of *ght5* gene under derepressed condition at 0.5 mM iron concentration, but slightly downregulated at 1 mM according to untreated control. But under repressed conditions, as iron concentrations increased, *ght5* transcription levels were gradually downregulated (Fig. 4). 2 mM iron concentration condition were excluded from RT-qPCR study due to the high toxicity on the cells.

Iron stress clearly downregulated *ght5* expression under derepressed condition, where expression level of *ght5* is expected to be higher. And under glucose repression, increasing iron stress also had a clear effect, which is in concordant with our previous report in which transcription of *ght5* was significantly downregulated with increasing iron concentrations (Ozkan et al. 2019).

### Effects of iron stress on *ght5* protein levels

**Figure 5.**
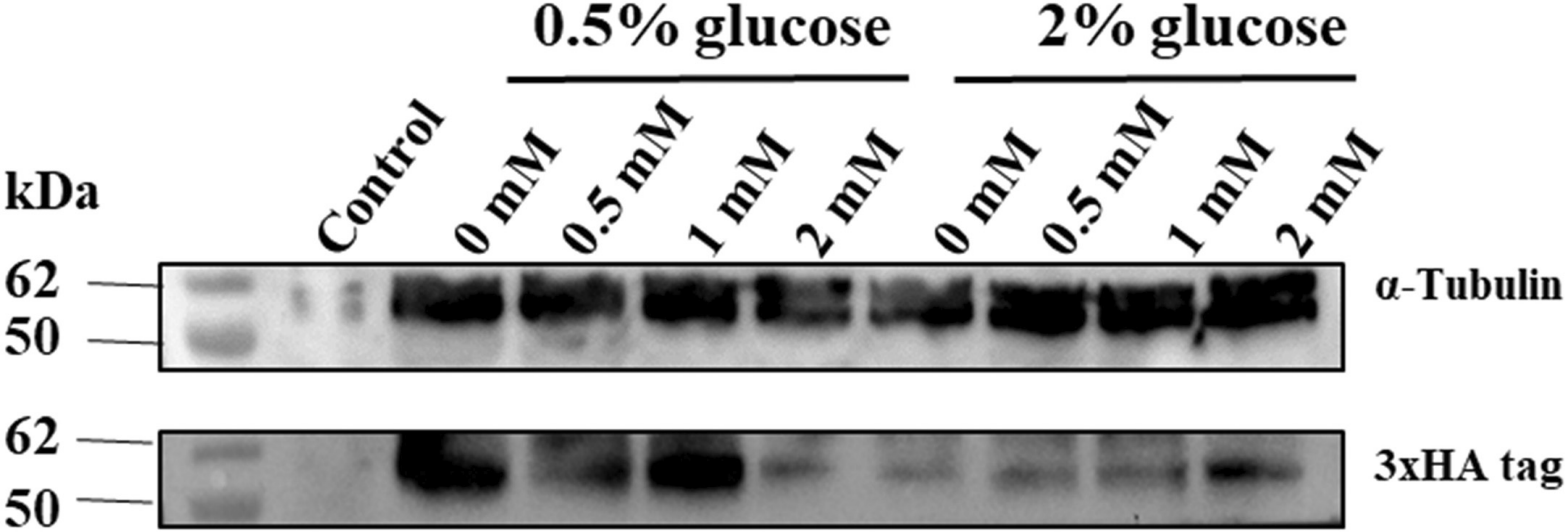
The expression of *ght5* in translation level analyzed by western blot. Western blot results of Ght5 under repressed and derepressed condition, containing different concentrations of iron. Cultures for protein extraction were prepared as indicated in total RNA isolation. Total protein extracts were used for all samples, and alpha-tubulin was used as reference protein. Lane 1: Untreated *S. pombe* ED666 cells under repressed condition (negative control). Lane 2–5:SA03 cells under derepressed condition treated with 0, 0.5, 1, 2 mM iron. Lane 6–9: SA03 cells under repressed condition treated with 0, 0.5, 1, 2 mM iron.

Protein levels of Ght5 were examined by western blot. The C-terminal 3xHA tagged Ght5 protein was analyzed by immunoblotting with anti-HA antibody in SA03 cells treated with iron (0, 0.5, 1, and 2 mM) under both derepressed (0.5% glucose) and repressed (2% glucose) conditions. In addition, anti-alpha tubulin antibody was used for normalization. And the results showed that each line contained a similar protein concentrations for anti-tubulin antibody. *S. pombe* ED666 parental cells were used as negative control for anti-HA antibody (Fig. 5).

The results of translation levels under iron stress conditions were inconsistent, as expected, due to the instability of episomal plasmid. In contrast with RT-qPCR, with the help of C-terminal 3xHA epitope tag, western blot can directly show the expression of Ght5, which is expressed only from the episomal multicopy plasmid. We can clearly see that in untreated SA03 cells, Ght5 protein content was significantly higher in derepressed condition than that of repressed condition, and this is in concordance with previously reported results (Saitoh et al. 2015).

Even though inconsistencies of the expression data from the episomal plasmid are limitations of this study, we can still follow the levels and localization of cytoplasmic Ght5 protein.

### Effects of iron on subcellular localization of ght5

Understanding the effects of iron stress on the subcellular localization of Ght5 is important in helping to understand both the function of Ght5, and the organization of the cell as a whole. Therefore, SA04 cells (cells containing pSA04 plasmid) were used to analyze the subcellular localization of Ght5 hexose transporter protein in *S. pombe*. As we have seen that the episomally expressed Ght5 protein levels were significantly higher under derepressed condition, the effects of iron stress on Ght5 localization were investigated under 0.5% glucose (derepressed) concentrations.

**Figure 6.**
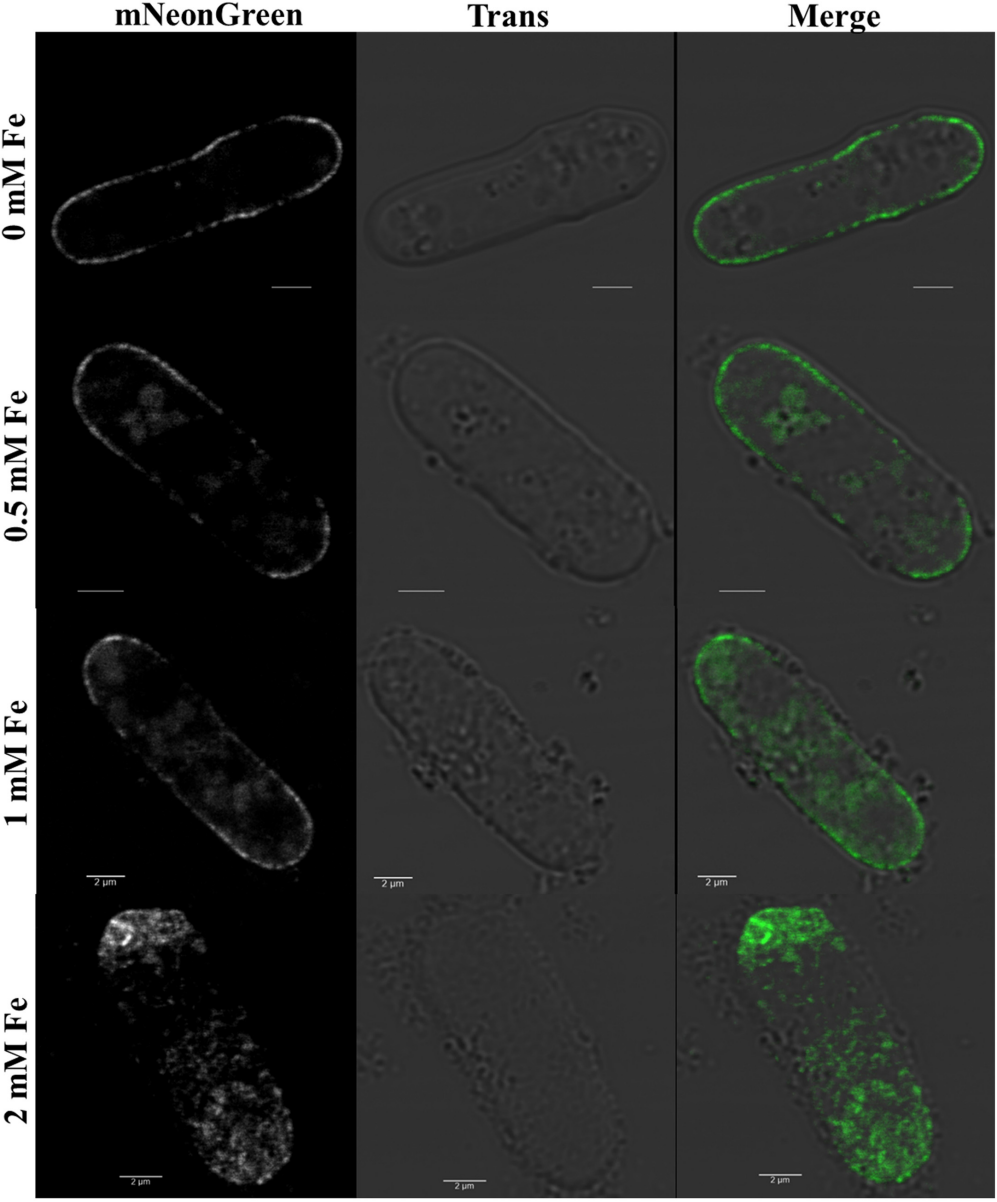
Subcellular localization of Ght5 under iron stress. Transformed *S. pombe* cells with pSA04 plasmis were used. Cells were grown until mid-log phase in minimal medium containing 0.5% glucose, and increasing iron concentrations (from top to bottom). Imaging was done by confocal microscopy.

Membrane localization of Ght5-mNeonGreen fusion protein is decreased dependent on increased iron stress, whereas cytoplasmic accumulation of the protein was increased (Fig. 6). Ozkan et al. (2019) showed that glucose consumption is lowered under iron stress. These results are also in parallel with our findings of cell viability assays in which viability was decreased with iron stress.

Ght5 is known as the essential and mostly expressed hexose transporter in *S. pombe* under low glucose conditions (Saitoh et al. 2015). TORC2-Gad8/AKT pathway ensures glucose uptake and proliferation, it is shown to be in charge of the localization of Ght5 on the cell surface by blocking Aly3 dependent endocytosis of Ght5 in the presence of nitrogen (Toyoda et al. 2021). Considering this, effect of iron stress on Ght5 localization might be related with TORC2-Gad8/AKT pathway.

Herein, we showed for the first time that the localization of Ght5 on the cellular membrane was decreased dependent on the increasing iron stress.

## Conclusion

Ght5 is the essential hexose transporter under low glucose condition. We showed that iron stress downregulated the transcription of *ght5*, and decreased the amount of Ght5 protein that are localized on the cell surface. This possibly results in decreased glucose consumption as well as decreased cell viability. Other than its toxicity, excess iron might have a secondary inhibition effect on cellular growth by modifying membrane localization of Ght5.

In this study, we suggest that iron stress might have a relation with regulation of Ght5 localization for the first time. This relation should be thoroughly investigated.
